# Supplementary material for: Toxicity of TiO2, SiO2, ZnO, CuO, Au and Ag engineered nanoparticles on hatching and early nauplii of Artemia sp
Source: PeerJ. 2019 Jan 3;6:e6138. doi: 10.7717/peerj.6138 (PMC6321756; doi:10.7717/peerj.6138)
Supplement: Supplemental Information 1 — Figure S1 The pattern of distribution of particles in MilliQ and salt water for three days. DLS measurements were made up to 48 h. We show an increase in aggregates size for some of the nanoparticles. Except for TiO2 and SiO2; most of the particles remain suspended and do not show visible sediment. Figure S2 Images of Artemia hatched in presence of nanoparticles a. control, b, g-silver nanoparticles, c-CuO nanoparticles. Nauplii do not show any gross abnormality but often one sees still-dead nauplii enclosed in the membrane (e, f) where appendages are not free. Figure S3 Artemia hatched in the presence of nanoparticles, were stained for reactive oxygen species using DCFDA, and Dead nauplii showed the staining in the gut (V), and the whole body in still–dead unhatched nauplii with membrane [C–F]. Supplementary table Data of individual experiments for hatching of Artemia in presence of SiO2NP. At 0.011 mg/L concentration the percent hatching of Artemia was always more as compared to hatching in salt water. (data not presented in the paper) [file peerj-07-6138-s001.docx]

**Supplementary material**

**
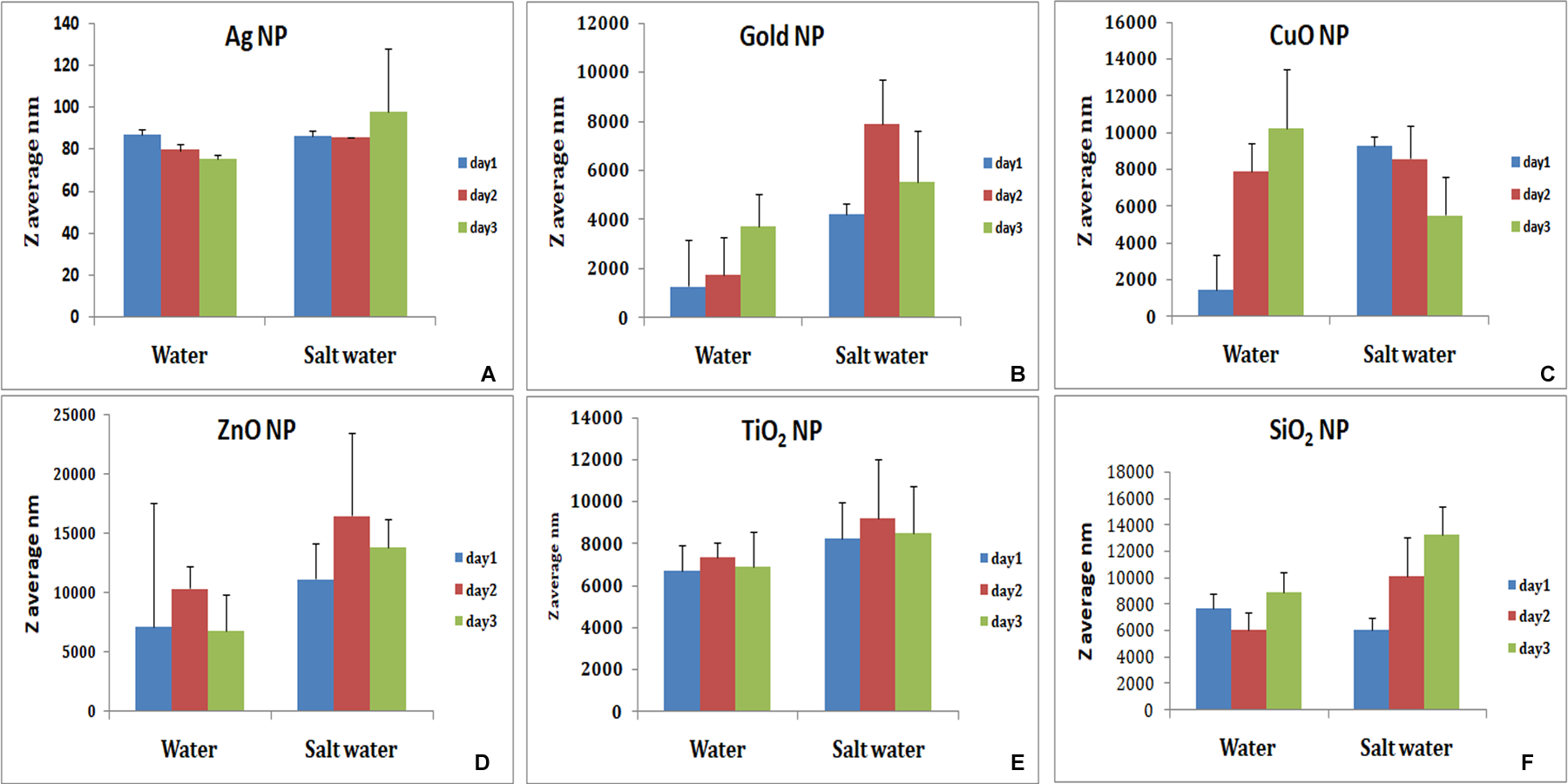
**

**Figure S1** The pattern of distribution of particles in MilliQ and salt water. DLS measurements were made up to 48 h. We show increase in aggregates size for some of the nanoparticles. Except TiO_2_ and SiO_2_; most of the particles remain suspended and do not show visible sediment.

**
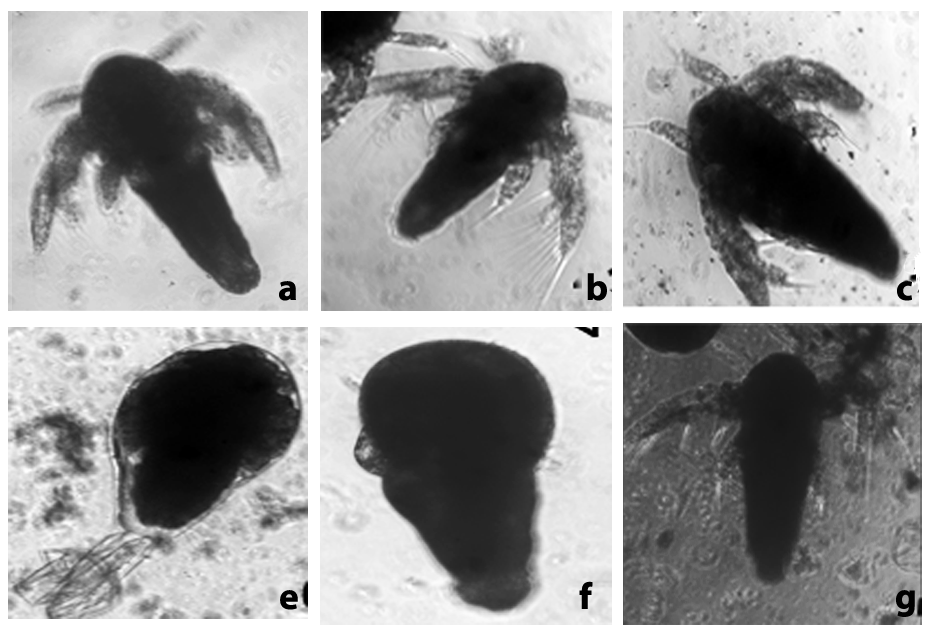
**

**Figure S2** Images of *Artemia* hatched in presence of nanoparticles; a - control, b, g -silver nanoparticles, c-CuO nanoparticles. Nauplii do not show any gross abnormality but often one sees still-dead nauplii enclosed in the membrane (e, f) where appendages are not free.

**
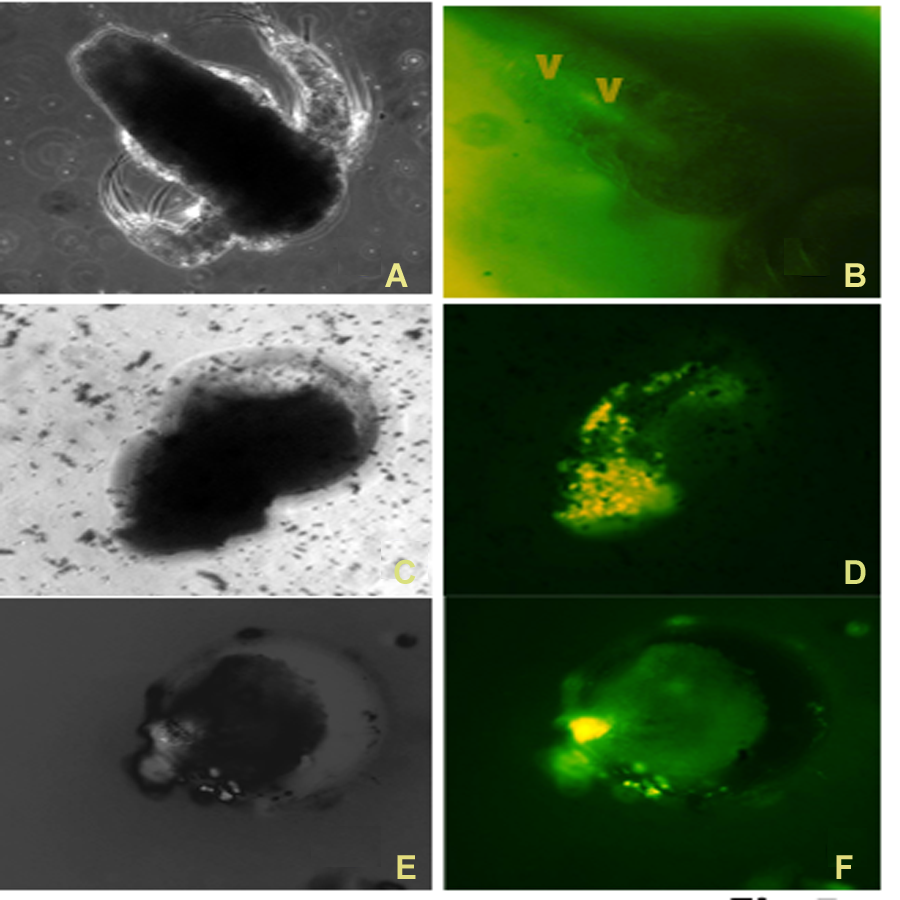
**

**Figure S3** *Artemia* hatched in the presence of silver nanoparticles, were stained for reactive oxygen species using DCFDA, and dead nauplii showed the staining in gut (V), and whole body in stilldead unhatched nauplii within the membrane [C-F].

Supplementary Table 1

Percent hatching of *Artemia* in presence of SiO_2_ nanoparticles. Data of individual experiments to show that at lower concentrations of SiO_2_NPs the hatching rate was higher.

|  | Percent hatching | Percent hatching | Percent hatching | Percent hatching | Percent hatching | Percent hatching | Percent hatching | Mean percent hatching | SD |
| --- | --- | --- | --- | --- | --- | --- | --- | --- | --- |
| blank | 50 | 97.974 | 71.9 | 70 | 75 | 49.46 | 79.19 | 70.5034 | 16.910 |
| SiO2-100mg/L | 44.14 | 79.9633 | 58.8 | 50 | 30 | 11.76 | 60.86 | 48.5638 | 22.210 |
| SiO2-10mg/L | 80.5 | 91.8233 | 80.5 | 82 | 28.85 | 45.45 | 74.64 | 67.2105 | 22.9229 |
| SiO2-1mg/L | - | 89.46 | 91 | 86.6 | 77.32 | 63.4 | - | 81.556 | 11.453 |
